# Diversity of Endophytic Fungi of the Coastal Plant *Vitex rotundifolia* in Taiwan

**DOI:** 10.1264/jsme2.ME18075

**Published:** 2019-02-05

**Authors:** Yu-Hung Yeh, Roland Kirschner

**Affiliations:** 1 Department of Life Sciences, National Central University No. 300, Zhongda Rd., Zhongli District, Taoyuan City 32001 Taiwan (R.O.C.); 2 Department of Biomedical Sciences and Engineering, National Central University No. 300, Zhongda Rd., Zhongli District, Taoyuan City 32001 Taiwan (R.O.C.)

**Keywords:** *Acrophialophora*, halophytes, *Phyllosticta capitalensis*, sand coast dune ecology

## Abstract

*Vitex rotundifolia* L. f. (Lamiaceae), which commonly grows at sand coasts, is important for coast protection and the prevention of erosion. However, the diversity and roles of fungi associated with this plant remain unclear. A total of 1,052 endophytic isolates from 1,782 plants tissues from two sand beaches in northern Taiwan were classified into 76 morphospecies based on culture morphology and ITS or LSU rRNA gene sequence comparisons. Critical species were further identified using protein gene sequences and microscopy. Most of the isolates at both sites belonged to the phylum Ascomycota, with Pleosporales having the most species (15 species). The largest number of isolates (47.7%) was from the stems, followed by the roots (22.5%), leaves (16.6%), and branches (13.1%). The three species with the highest isolation frequencies at both sites were *Alternaria alternata*, *Aspergillus terreus*, and an undescribed species of *Alpestrisphaeria*. *A. terreus* was found in all organs. *A. alternata* was detected in all organs, except the roots. *Alpestrisphaeria* sp. was only found in the roots and stems. In the stems and roots, strain numbers from cortical tissues were approximately two-fold higher than those from the corresponding woody tissue. The overall colonization rate in the stems was significantly higher than those that in the roots and leaves. The majority of fungi appeared to be saprobes, which may play important roles in nutrient recycling during sand burial and mediate further stress factors in the coastal habitat.

Endophytes are microorganisms, mainly fungi, that grow within plant tissues for all or part of their life cycle without causing any apparent disease (30). Endophytes are important components of fungal biodiversity. Approximately 100,000 fungal species have been identified to date, comprising *ca*. 7% of the estimated 1.5 million species on this planet (8). This estimation is also due to the fact that fungal endophytes have been isolated from all plant species investigated so far. Over the course of evolution, endophytes and their host plants have established specific relationships. Based on their interactions with host plants, endophytic fungi may be divided into three groups: mutualists, parasites, and commensalists (10, 12). In mutualism, certain endophytic fungi confer tolerance to abiotic and biotic stresses on their host plants, enhance their growth, and suppress diseases (18, 19). The roles of most endophytes in their host plants currently remain unclear.

Endophytic fungi are also major potential sources for bioactive compounds and drug development (10). After the discovery that an endophytic fungus isolated from the bark of *Taxus brevifolia* had the ability to produce taxol (35), the number of studies on endophytes significantly increased. The identification of a species of endophytic fungi in a given plant is a fundamental step towards addressing their diversity and potential roles in the plant as well as for the discovery of novel bioactive compounds. Since many endophytic isolates grow as sterile mycelia, they cannot be identified by morphology alone. Due to the lack of DNA data for approximately 80% of known fungal species and the limitations associated with the internal transcribed spacer (ITS) sequences as barcodes in mycology (15, 21), the sole use of molecular identification is not feasible.

In traditional medicine, the fruit of *Vitex rotundifolia* L. f. (Lamiaceae) is used to treat headache, cold, eye pain, asthma, chronic bronchitis, and some allergic diseases (9, 24). *V. rotundifolia* commonly grows at sand coasts and tolerates high salinity, wind and sand movements, and lack of nutrients (Fig. 1). Due to its capacity for the regenerative growth of new stems and leaves after sand burial, this plant is important for coast protection and the prevention of erosion. *V. rotundifolia* has a wide natural range in America, Asia, and Australia. In the southeastern United States, *V. rotundifolia* is an invasive species that is a major threat to fragile coastal dune ecosystems (3). This plant contributes to major coastal vegetation in Taiwan, which is endangered by construction, tourism, and other human activities, as well as rising sea levels. To date, only five species of pathogenic fungi have been recorded from *V. rotundifolia* worldwide (Farr, D.F., and A.Y. Rossman. Fungal Databases, U.S. National Fungus Collections, ARS, USDA. Retrieved May 9, 2018, from https://nt.ars-grin.gov/fungaldatabases) (33), and the endophytic fungi associated with this plant have not yet rarely been investigated. The purpose of the present study was to detect and identify endophytic fungi from different organs of *V. rotundifolia* in order to elucidate their potential roles in the plant.

## Materials and Methods

### Plants and collection sites

Between 2013 and 2015, individuals of *V. rotundifolia* were collected at two sandy sites of the northern coast of Taiwan (Taoyuan 25.047°N 121.076°E, Hsinchu 24.765°N 120.911°E). Three plants were collected from two beaches in two counties of Taiwan per sampling (a total of three times). Plants that were not conspicuously buried by sand during the collection time were removed with a trowel, individually placed in bags, returned to the laboratory, and kept at 4°C until further processing. Plants were processed for endophyte isolation within 48 h of sampling.

### Isolation of fungi

Samples were washed and divided into roots, stems, leaves, and branches. Three roots, stems, and branches as well as six leaves per each of the three collected plants were randomly selected. Plant fragments were surface-sterilized under sterile conditions by agitation in 95% ethanol for 1 min, 6–12% sodium hypochlorite (5% chloride) for 3 min (for stems and branches) or 1.5 min (for roots and leaves), 95% ethanol for 0.5 min, rinsed in sterile water, and allowed to dry for a few min. The effectiveness of surface sterilization was tested by making imprints of plant fragments on malt extract agar (MEA) plates (19). Each root and stem segment was divided into cortical tissues and wood by peeling the cortical tissues from the wood and cutting all parts into 12 segments of approximately 1–2 cm, each branch was cut into three segments of approximately 1–2 cm, and each leaf into the petiole as well as two segments of *ca*. 0.6 cm in diameter from the lamina. These tissue segments were immediately placed onto MEA with 0.2% chloramphenicol. All isolates obtained from each plant sample were classified according to their morphological appearance into morphotypes. Representative isolates were investigated and deposited at the Bioresource and Collection Center, Hsinchu, Taiwan (BCRC). Dried cultures were deposited at the herbarium of the National Museum of Natural Science, Taichung, Taiwan (TNM, Table S1).

### Molecular identification

Total genomic DNA isolation, PCR, and sequencing were performed as described by Yeh & Kirschner (32). PCR products were amplified with different primers to identify species. Primer selection was based on published phylogenetic analyses of specific genera of fungi. The DNA sequences of the ITS and/or ribosomal large subunit RNA gene (LSU rDNA) were generated for preliminary identification. Several genera required an additional barcode. The primer pair TUB2Fd/TUB4Rd was used for amplification of the beta tubulin gene (7), particularly for the identification of *Colletotrichum* species (4, 28), *Diaporthe* species (6, 26), and *Aspergillus* species (21). Partial sequences of the gene coding for Histone H3 (HIS3) were generated for *Fusarium* species (2). Regarding *Phyllosticta* species, partial sequences of the actin (act) gene were generated (29). The glycerol-3-phosphate dehydrogenase gene (GPDG) was additionally applied for strains of *Curvularia* (14). DNA sequencing was performed by Mission Biotech (Nankang, Taipei) with the same primers used for PCR. DNA sequences were edited using CodonCode Aligner version 4.0.1 (CodonCode, Centerville, MA, USA) and submitted to GenBank and the DNA Data Bank of Japan (Table S2). Sequences were submitted to BLAST searches at GenBank (https://blast.ncbi.nlm.nih.gov/). Depending on similarities in the barcodes used and verifiability by morphology, strains were identified as species, genus, or higher taxonomic ranks. Only sequences cited in publications were considered for identification. The scientific names retrieved from GenBank were updated with Index Fungorum (http://www.indexfungorum.org).

### Morphological study

Microscopy was used to verify DNA-based identities in groups, in which DNA data were suspected to be misleading because of the lack of data or insufficient resolution. Regarding light microscopy, specimens from cultures were mounted in 10% (w/v) KOH solution. For example, the morphological world monograph by Simmons (23) was used to identify *Alternaria* strains. The identification of *A. terreus* was confirmed by the detection of accessory conidia (11, 20). In *Alpestrisphaeria* sp., the discovery and morphological characterization of a *Monodictys* anamorph supported its distinction from all known species in both genera (it will be described in another publication).

### Statistical data analysis

**Fig. 2:**
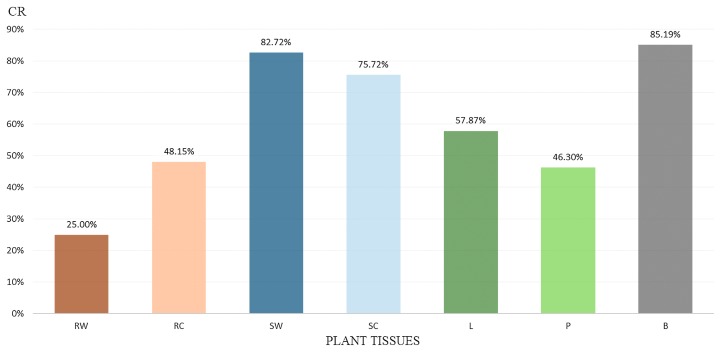
Colonization rate of different plant tissues from *Vitex rotundifolia*. RW: root wood, RC: root cortical tissues, SW: stem wood, SC: stem cortical tissues, L: leaf lamina, P: petiole, B: branch.

Isolation frequency was defined as the percentage of tissue segments yielding an endophyte in culture. The isolation frequency of fungal species was calculated using the following formula: IF=Ni/Nt×100, where Ni is the number of segments from which a given species is isolated and Nt is the total number of segments used for isolation (Table S1). The colonization rate was defined as the percentage of all fungal species in culture (Fig. 2), which was calculated using the following formula: CR=Nc/Nt×100, where Nc is the total number of segments from which any fungus is isolated, and Nt is the total number of segments used for isolation (25).

## Results

A total of 1,052 endophytic isolates were obtained from 1,782 plants tissues and divided into 76 morphospecies based on culture macromorphologies and ITS or LSU sequence comparisons. Species were ordered according to their frequencies in Table S1, and based on their systematic positions in Table S3. Critical species were further identified using protein gene sequences and microscopy. Forty-four species were found at Taoyuan and 45 at Hsinchu, while only 13 were detected at both sites. The majority of isolates at both sites belonged to the phylum Ascomycota, 4 to Basidiomycota, and only 1 to Zygomycota (Table S3). Among Ascomycota, 13 species were incertae sedis, whereas all other species belonged to 18 orders, with Pleosporales having the most species (15 species). The largest number of isolates (47.7%, 49 species) were from stems, followed by roots (22.5%, 27 species), leaves (16.6%, 31 species), and branches (13.1%, 27 species). The three species with the highest isolation frequencies at both sites were *A. alternata* (204 strains, IF 11.45%; 133 from Hsinchu, IF 7.45%; 71 from Taoyuan, IF 4%), *A. terreus* (165 strains, IF 9.26%), and *Alpestrisphaeria* sp. (159 strains, IF 8.92%). *A. terreus* was found in all organs, but was approximately two-fold more frequent in the winter season (November to February, 109 strains) than in the summer season (August, 56 strains) (Table S4). *A. alternata* was found in all organs, except for the roots, and was more frequent in the winter season (192 strains) than in the summer season (12 strains). *Alpestrisphaeria* sp. was only found in the roots and stems and only in the winter season (Table S4). In the stems and roots, strain numbers from cortical tissues were approximately two-fold higher than those from the corresponding woody tissue. The overall colonization rate in the stems was significantly higher than that in the roots and leaves; the overall colonization rate of the branches was slightly lower than that of the stems. Regarding different plant tissues, endophytic fungal communities had varying degrees of overlap. In the stems, 23 species (45.1%) were isolated from both the central and cortical tissues. Endophytic fungal communities were the most diverse in the leaves, with only 7 (21.2%) species being found in the petiole and lamina. Most species (56 species) were represented by less than 9 strains; however, some were common only at one of both collection sites. The most common of these species was *Acrophialophora* sp. 2, which was only found at Hsinchu (a total of 84 strains, IF 4.71%). Other common species were *Acrophialophora* sp. 3 and *Phyllosticta capitalensis*.

## Discussion

In the present study, 76 discovered fungal species were new records for this host, and were arranged according to their frequencies. Therefore, their potential roles in natural populations of *V. rotundifolia* remain unknown. *A. terreus* is a widely distributed fungus and was one of the most frequently detected fungi in the present study. *A. terreus* is only exceptionally reported as a plant pathogen, but may contribute to the resistance of the host plant to diseases (27). The roles of the other most common species found in the roots and stems may be saprobic or mutualistic rather than parasitic. *A. alternata* was one of the most frequent fungi in the present study and the most common species in the leaves. It was also the most common endophyte in the halophilic plant *Salicornia europaea* in Japan (16). In *V. rotundifolia*, this fungus may play an important role in nutrient recycling, particularly when leaves die due to sand burial, decompose, and are replaced by leaves of the newly grown shoot apex. This nutrient recycling may be particularly significant in the coastal habitat of halophytes with relatively few soil nutrients and frequent sand burial. *Ph. capitalensis*, another common species mostly limited to leaves in the present study, may play a mutualistic role because, in contrast to most of the other *Phyllosticta* species, *Ph. capitalensis* is more frequently detected as an endophyte than as a leaf pathogen (29). Furthermore, no other *Phyllosticta* species were isolated in the present study and typical pathogenic species, such as *Fusarium*, *Curvularia*, and *Colletotrichum*, had low isolation frequencies. The habitat appears to be important as well as the plant species with respect to the composition of endophytic species (19). However, only a few species may be regarded as typical of coastal habitats, such as *Deniquelata barringtoniae*, and the basidiomycetous yeast *Rhodotorula paludigena*, or are known to grow in marine habitats, including *A. fumigatus*, *A. terreus*, and *Aureobasidium pullulans* (13, 34). Among the more than 1,000 endophytic strains from the natural habitat, we did not isolate *Corynespora cassiicola*, but detected this species as a leaf pathogen in an artificial plantation of *V. rotundifolia* in a botanical garden (33). Few potentially pathogenic species were identified, such as *F. oxysporum* and species of *Diaporthe*; however, their pathogenicities are largely strain-dependent and may only be evaluated by infection experiments. In the present study, the most common species were more frequently isolated from cortical than from inner tissues. *Acrophialophora* sp. 3 was exclusively found in cortical tissues. Since the imprint technique indicated the high efficacy of surface disinfection, the high isolation frequency from cortical tissues may not be caused by contamination, but rather appears to be based on higher nutrient availability than that for wood tissues. Studies on endophytic fungi rarely distinguish between fungi from the cortical and inner tissues of stems and roots, except for Fisher & Petrini (5), who also found a higher diversity of fungi from the whole stem than from xylem. Additionally, *Acrophialophora* is considered to be a thermotolerant soil fungus that widely exists in temperate and tropical regions as a decomposer (21). Therefore, we suggest that the study of endophytic non-mycorrhizal mutualistic interactions with a focus on bark and leaf tissues will provide a quick overview on fungal diversity.

*Alpestrisphaeria* sp. is a new species that was identified by morphology and DNA in the present study. This fungus did not yield a high BLAST similarity with ITS sequences in GenBank and was assigned to *Alpestrisphaeria* by LSU sequences. It will be described in a separate study. The limited information available on the two other known species in this genus (1, 36) indicate saprobic growth in soil and on dead plants. The densely aggregated melanized cells of the *Monodictys*-like anamorph found in cultures is typical of Dothideomycetes growing under stress conditions, such as high salinity, irradiation, or temperature (16).

Therefore, we concluded that most of the endophytes of *V. rotundifolia* were saprobic or mutualistic, but rarely parasitic, and may play important roles in mediating stress in the coastal habitat or in nutrient cycling after the death of plant parts by senescence or sand burial.

Although culture-independent approaches are very commonly applied in research on fungal diversity, these approaches have severe limitations in mycology in contrast to their application in prokaryotes. The ITS sequences of ribosomal RNA genes are regarded as a common barcode for fungi; however, data are only available for *ca*. 15.5% of known species (8, 22). In addition, up to 20% of fungal ITS sequences in databases are labeled with wrong species names (15). In many genera, including the most common molds, *e.g. Alternaria*, *Aspergillus*, and *Fusarium*, ITS sequences do not provide sufficient resolution for discrimination at the species level, and other genes are additionally required. In the present study, *A. alternata* and *A. arborescens* showed different morphologies, but not ITS sequences. In contrast to theoretical expectations, parallel cultivation-dependent and cultivation-independent approaches in the same study of fungi in sand dune vegetation found a small overlap of the species detected in both approaches (17, 31). The application of sophisticated statistical analyses to the limited data of either approach alone is questionable.

## Supplementary Information











## Figures and Tables

**Fig. 1:**
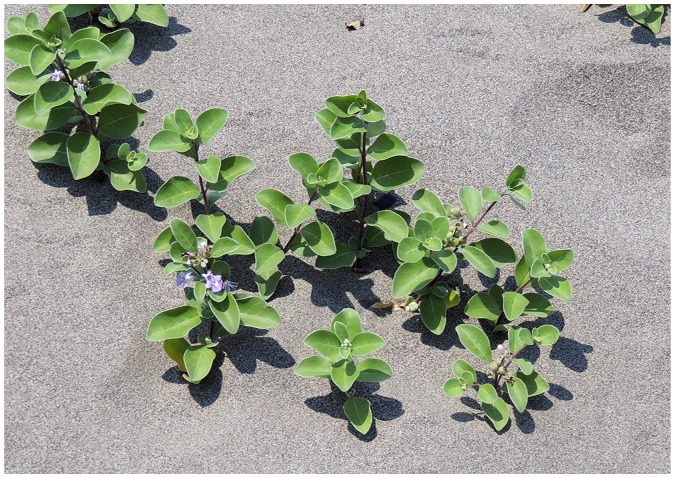
*Vitex rotundifolia* flowering under sand burial conditions (Taichung, Daan beach, Taichung City, Taiwan, 3^rd^ Sept. 2014).
