# Supplementary material for: Diversity of Endophytic Fungi of the Coastal Plant Vitex rotundifolia in Taiwan
Source: Microbes Environ. 2019 Feb 5;34(1):59–63. doi: 10.1264/jsme2.ME18075 (PMC6440724; doi:10.1264/jsme2.ME18075)
Supplement: Supplementary file 1 [file 34_59_s1.docx]

Supplements

**Table S1**. Origins, numbers and names of strains arranged according to isolation frequencies isolated from *Vitex rotundifolia*. RW: root wood, RC: root cortical tissues, SW: stem wood, SC: stem cortical tissues, L: leaf lamina, P: petiole, B: branch.

**Table S2**. DNA sequence and strain accessions of strains arranged according to strain numbers. ITS: internal transcribed spacer, LSU: large subunit ribosomal RNA gene, HIS: histone 3 gene, TUB: beta tubulin gene, TEF: translation elongation factor 1 alpha gene, ACT: actin gene, GPDH: Glycerol-3-phosphate dehydrogenase gene, BCRC: strain accession number of Bioresource Collection and Research Center, Hsinchu, Taiwan.

**Table S3**. Arrangement of endophytic fungi isolated from *Vitex rotundifolia* according to their systematic position (A = Ascomycota, B = Basidiomycota, Z = Zygomycota) and strain numbers from two different localities.

**Table S4**. Numbers of strains of endophytic fungi isolated from *Vitex rotundifolia* in the seasons winter (November to February) and summer (August) at the two localities Hsinchu and Taoyuan, Taiwan, arranged according to isolation frequencies.
